# Changes in fasting blood glucose status and incidence of cardiovascular disease: The China‐PAR project

**DOI:** 10.1111/1753-0407.13350

**Published:** 2023-01-13

**Authors:** Ye Tong, Fangchao Liu, Keyong Huang, Jianxin Li, Xueli Yang, Jichun Chen, Xiaoqing Liu, Jie Cao, Shufeng Chen, Ling Yu, Yingxin Zhao, Xianping Wu, Liancheng Zhao, Ying Li, Dongsheng Hu, Jianfeng Huang, Xiangfeng Lu, Chong Shen, Dongfeng Gu

**Affiliations:** ^1^ Department of Epidemiology, Center for Global Health School of Public Health, Nanjing Medical University Nanjing China; ^2^ Key Laboratory of Cardiovascular Epidemiology & Department of Epidemiology Fuwai Hospital, National Center for Cardiovascular Diseases, Chinese Academy of Medical Sciences and Peking Union Medical College Beijing China; ^3^ Tianjin Key Laboratory of Environment, Nutrition and Public Health, Department of Occupational and Environmental Health School of Public Health, Tianjin Medical University Tianjin China; ^4^ Division of Epidemiology Guangdong Provincial People's Hospital and Cardiovascular Institute Guangzhou China; ^5^ Department of Cardiology Fujian Provincial Hospital Fuzhou China; ^6^ Cardio‐Cerebrovascular Control and Research Center Institute of Basic Medicine, Shandong Academy of Medical Sciences Jinan China; ^7^ Sichuan Center for Disease Control and Prevention Chengdu China; ^8^ Department of Epidemiology and Health Statistics College of Public Health, Zhengzhou University Zhengzhou China; ^9^ Department of Epidemiology and Health Statistics School of Public Health, Shenzhen University Health Science Center Shenzhen China; ^10^ Research Units of Cohort Study on Cardiovascular Diseases and Cancers Chinese Academy of Medical Sciences Beijing China; ^11^ School of Medicine Southern University of Science and Technology Shenzhen China

**Keywords:** cardiovascular disease, Chinese population, impaired fasting blood glucose, prospective cohort study, 心血管疾病, 中国人群, 空腹血糖受损, 前瞻性队列研究

## Abstract

**Background:**

The effect of long‐standing prediabetes or its transition on incident cardiovascular disease (CVD) is unclear. This study aimed to evaluate the association of changes in fasting blood glucose (FBG) status with the risk of developing CVD.

**Methods:**

This research included 12 145 Chinese adults aged 35–74 years and free from diabetes mellitus (DM) at baseline. Study participants were cross‐classified into six categories according to glucose at the first (1998–2001) and the second visit after 8 years: normal fasting glucose (NFG; 50–99 mg/dl), impaired FBG (IFG; 100–125 mg/dl), and DM. Cox proportional hazard regression model was used to estimate the hazard ratio (HR) and 95% confidence interval (CI) for CVD associated with transition of glucose status.

**Results:**

During a median follow‐up of 5.5 years, 373 incident CVD cases occurred. Compared with participants remaining persistent NFG, a higher risk of developing CVD was identified among those remaining persistent IFG, progressing to DM from NFG or from IFG, with the multivariate‐adjusted HR (95% CI) of 1.792 (1.141, 2.816), 1.723 (1.122, 2.645) and 1.946 (1.120, 3.381), respectively. Furthermore, when stratified by glucose status at baseline, persistent IFG and progression from IFG to DM still increased CVD risk in comparison with reversion from IFG to NFG, with the multivariate‐adjusted HR (95% CI) of 1.594 (1.003, 2.532) and 1.913 (1.080, 3.389).

**Conclusions:**

Participants with long‐standing IFG and progressing to DM had a higher risk of developing CVD. Further well‐designed studies are warranted to assess the association of other phenotypes or prediabetes duration with CVD.

## Graphical abstract

**Highlights**

- Adjusted for age, gender, BMI, FBG at the first visit, smoking status (never smoker, former smoker, current smoker), drinking status (yes vs no), physical activity, hypertension status (yes vs no), dyslipidemia (yes vs no), region (south vs north), area (urban vs rural), education, income and family history of CVD.
- BMI, body mass index; FBG, fasting blood glucose; NFG, normal fasting glucose (50‐99 mg/dL); IFG, impaired fasting glucose (100‐125 mg/dL); DM, diabetes mellitus (≥126 mg/dL and/or using hypoglycemic drugs, and/or a self‐reported history of DM); CVD, cardiovascular disease.

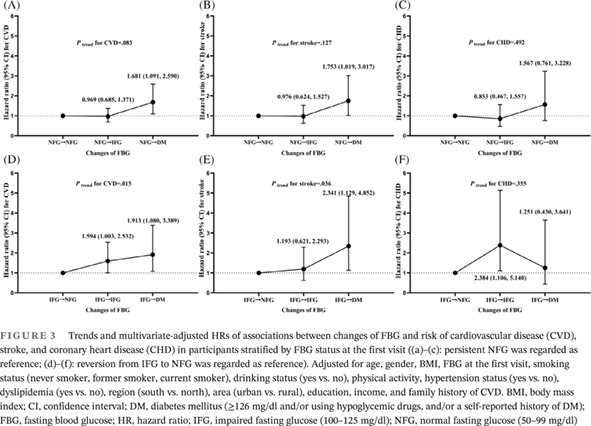

## 摘要

**背景:**尚不清楚长期存在的糖尿病前期或其转变对心血管疾病(CVD)事件的影响。本研究旨在评估空腹血糖(FBG)状态变化与CVD发生风险之间的关系。

**方法:**本研究纳入了12145名35‐74岁、基线时无糖尿病(DM)的中国成年人。研究参与者根据第一次(1998‐2001年)和8年后第二次就诊时的血糖将其交叉分为6类:正常空腹血糖(NFG, 50‐99 mg/dL)、空腹血糖受损(IFG, 100‐125 mg/dL)和糖尿病。采用Cox比例风险回归模型估计CVD与葡萄糖状态转变相关的风险比(HR)和95%置信区间(CI)。

**结果:**在中位随访5.5年期间，共发生373例CVD病例。与持续存在NFG的参与者相比，在持续存在IFG、从NFG或IFG进展为DM的参与者中，发生CVD的风险更高，多变量校正HR (95% CI)分别为1.792(1.141,2.816)、1.723(1.122,2.645)和1.946(1.120,3.381)。此外，当按基线血糖状态分层时，与从IFG逆转到NFG相比，持续IFG和从IFG进展到DM仍增加CVD风险，多变量校正HR (95% CI)分别为1.594(1.003,2.532)和1.913(1.080,3.389)。

**结论:**长期存在IFG并发展为糖尿病的参与者发生CVD的风险更高。需要进一步精心设计研究，评估其他表型或糖尿病前期持续时间与CVD的相关性。

## 1 INTRODUCTION

Prediabetes is a chronic condition that occurs when blood glucose is elevated above the normal range but below the diabetes diagnostic threshold,^1^ defined as impaired fasting glucose (IFG, fasting plasma glucose of 100–125 mg/dl) and/or impaired glucose tolerance (IGT, 2‐h plasma glucose of 140–200 mg/dl during 75‐g oral glucose tolerance test) and/or elevated hemoglobin A1c (HbA1c, 5.7%–6.4%). The prevalence of prediabetes has been increasing over recent years.^2^ It is estimated that there will be more than 470 million people with prediabetes by 2030 worldwide.^3^ The China Chronic Disease and Risk Factors Surveillance, a nationally representative cross‐sectional survey in mainland China, showed that the prevalence of prediabetes in adults was 38.1% in 2018, which was characterized by increasing significantly in urban residents between 2013 and 2018.^4^ The number of adults with prediabetes is expected to be more than 196 million by 2045 in China,^2^ so there is a particularly urgent need to deal with the challenge.

Prediabetes signifies the risk of developing type 2 diabetes.^2^, ^5^ Diabetes is related to obesity, inactivity, and aging,^6^ and it is a risk factor of cardiovascular disease (CVD).^7^ Evidence also shows that prediabetes may be involved with reduction in insulin sensitivity, dysfunction of pancreatic beta cell, and further promotion of atherosclerosis.^8^ However, associations between prediabetes and CVD differed by prediabetes diagnostic criterion, type of CVD, and ethnicity,^9^ and the research was mostly based on single measurement of blood glucose,^10^, ^11^ so it was uncertain whether prediabetes itself or the progression of prediabetes to diabetes would increase the risk of CVD events. Findings from the Diabetes Prevention Program indicated that individuals reverting from prediabetes to normoglycemia experienced a reduction in their cardiovascular risk.^12^ This evidence, as a result, has spurred interest in the health impacts of changes in blood glucose status on incident CVD, and the potential mechanism has attracted the attention of researchers.

Several studies reported progression to diabetes rather than long‐standing prediabetes was associated with an increased CVD risk.^13^, ^14^, ^15^ However, study of an occupational cohort from Japan found that long‐standing prediabetes was associated with CVD when prediabetes was jointly defined by IFG and HbA1c, and the association was not significant when prediabetes is defined by IFG alone.^16^ Two prospective studies conducted in China focused on exploring the potential benefits of reversion from prediabetes to normoglycemia and long‐standing prediabetes on CVD when compared with progression to diabetes mellitus (DM).^17^, ^18^ However, prediabetes in these two studies was detected in different methods, and whether prediabetes itself instead of progression to DM induces CVD in Chinese population is unclear. Therefore, it is necessary to carry out further research of the association of long‐standing prediabetes with incident CVD in the Chinese population. Meanwhile, fasting blood glucose (FBG) is a simply measured, cheap and rapidly available method of detecting blood glucose level. Thus, figuring out the effect of changes of FBG on CVD might be efficient in CVD primary prevention.

In this study, we aimed to investigate the potential effect of changes in FBG on the incidence of CVD based on data from the Prediction for Atherosclerotic Cardiovascular Disease in China (China‐PAR) project and further evaluate whether long‐standing prediabetes increases CVD risk. It may provide evidence of long‐term blood glucose control for the primary prevention of CVD in the population without diabetes.

## 2 METHODS

### 2.1 Study population

Participants were from two cohorts of the China‐PAR project, the China Multi‐Center Collaborative Study of Cardiovascular Epidemiology (China MUCA‐1998), and the International Collaborative Study of Cardiovascular Disease in Asia (InterASIA). These two cohorts were established in 1998 and 2000–2001, respectively. Detailed description of study design was previously published.^19^, ^20^ The second survey of the China MUCA‐1998 and the InterASIA study was conducted from 2007 to 2008, and the third survey was conducted from 2012 to 2015.

**FIGURE 1:**
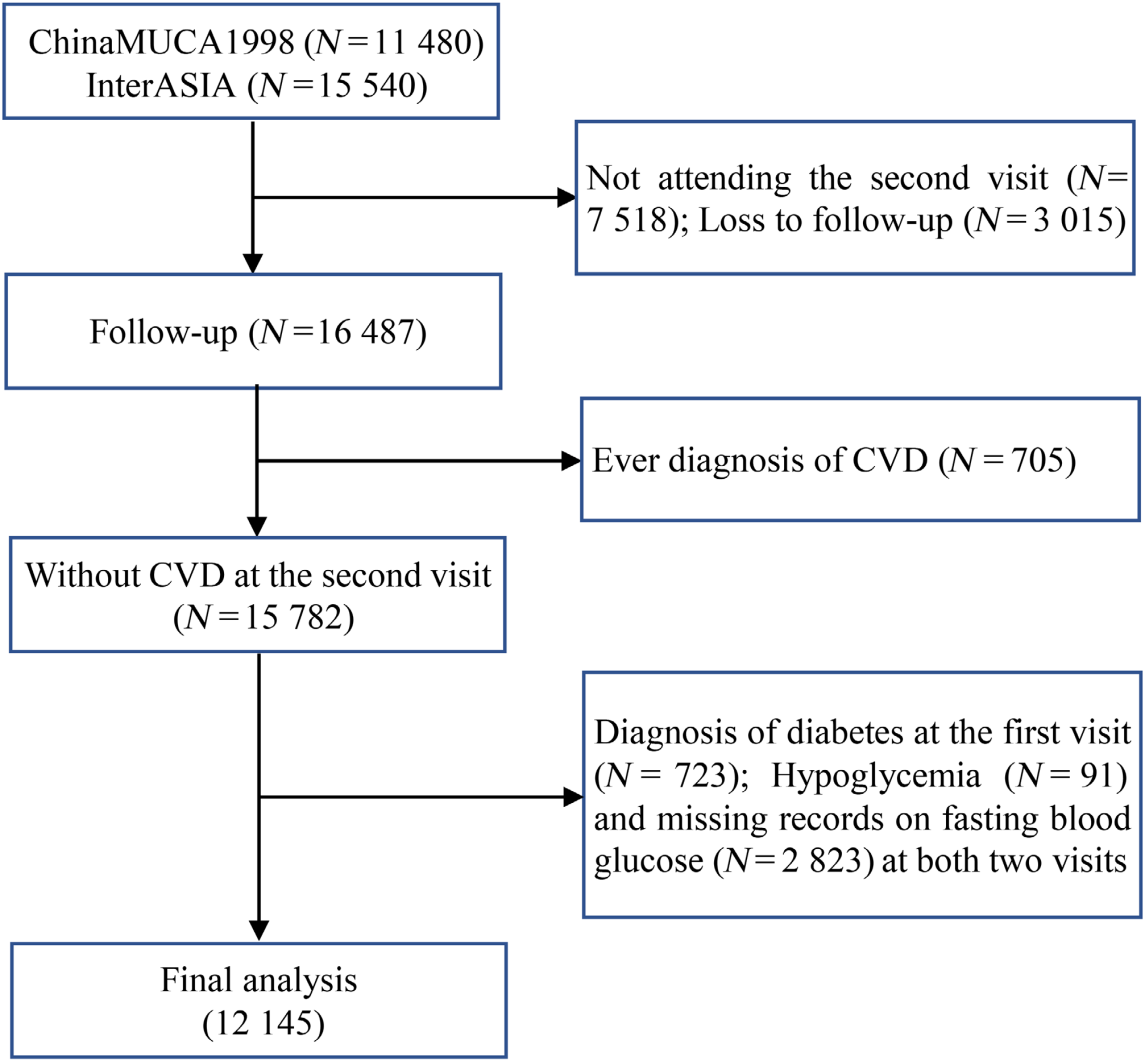
Flow chart of inclusion and exclusion of participants in the cohort study. CVD, cardiovascular disease; China MUCA, China Multi‐Center Collaborative Study of Cardiovascular Epidemiology; InterASIA, International Collaborative Study of Cardiovascular Disease in Asia

A total of 27 020 Chinese adults aged 35–74 years were enrolled at the first visit (*n* = 11 480 from China MUCA‐1998; *n* = 15 540 from InterASIA). Participants were excluded due to (1) absence of the second visit (*n* = 7518), of whom 1045 died; (2) loss to follow‐up (*n* = 3015); (3) the history of CVD (nonfatal myocardial infarction [MI], unstable angina, death from coronary heart disease [CHD], fatal or nonfatal stroke, heart failure) before the second visit (*n* = 705); (4) the diagnosis of diabetes at the first visit (*n* = 723); (5) hypoglycemia (<50 mg/dl, *n* = 91); and (6) missing records on glucose‐related information at both visits (*n* = 2823). Finally, altogether 12 145 subjects were included in the analysis (Figure 1). The Institutional Review Board at Fuwai Hospital in Beijing and all participating institutions approved this study. Informed consents were obtained from participants before data collection.

### 2.2 Assessment of FBG and category of changes in FBG

Blood samples were collected to measure serum glucose after fasting for at least 10 h, and were centrifuged immediately at 2000g for 10 min at 4°C or room temperature. Serum glucose concentrations were measured by a modified hexokinase enzymatic method (automatic clinical analyzer, Model 7060; Hitachi, Tokyo, Japan). Detailed information on blood specimen measurement is elaborated in Appendix S1.

**FIGURE 2:**
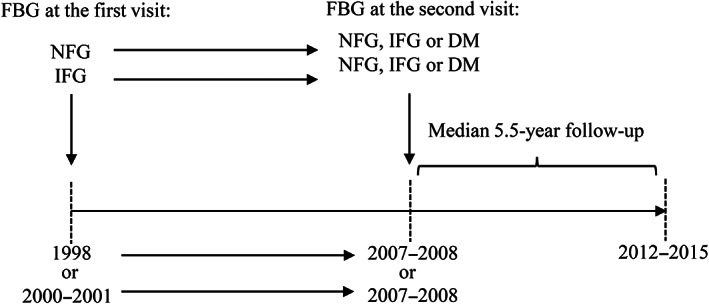
Timeline of the study design. Subjects free from diabetes mellitus (DM) at the first visit (1998 or 2000–2001) and without cardiovascular disease (CVD) before the second visit (2007–2008) were followed 8 years later for the following changes: (1) persistent NFG, (2) NFG to IFG, (3) NFG to DM, (4) IFG to NFG, (5) persistent IFG, and (6) IFG to DM. DM, diabetes mellitus (≥126 mg/dl and/or using hypoglycemic drugs, and/or a self‐reported history of DM); FBG, fasting blood glucose; IFG, impaired fasting glucose (100–125 mg/dl); NFG, normal fasting glucose (50–99 mg/dl)

Study participants were defined as follows based on blood glucose level: normal fasting glucose (NFG; 50–99 mg/dl), IFG (100–125 mg/dl), and DM (≥126 mg/dl and/or using hypoglycemic drugs, and/or a self‐reported history of DM).^1^, ^21^ According to blood glucose levels at both the first and the second visit, participants were classified into six groups: persistent NFG, progression from NFG to IFG, progression from NFG to DM, reversion from IFG to NFG, persistent IFG, and progression from IFG to DM (Figure 2), to examine the effect of changes in FBG status on CVD risk.

### 2.3 Assessment of covariates

Hypertension was defined as currently using antihypertensive medicine and/or an average of three sitting systolic blood pressure (SBP) measurements ≥140 mmHg and/or an average of three sitting diastolic blood pressure measurements ≥90 mmHg after at least a 5‐min rest and with a 30‐s interval. Total cholesterol (TC), triglyceride (TG), and high‐density lipoprotein cholesterol (HDL‐C) were assessed on a Hitachi 7060 Clinical Analyzer (Hitachi High‐Technologies Corp., Tokyo, Japan) with cholesterol oxidase method, glycerophosphate oxidase method, and direct chemically modified enzyme method, respectively.^22^ Low‐density lipoprotein cholesterol (LDL‐C) levels were calculated using the Friedewald formula (LDL‐C = TC − HDL‐C − TG/5) for the participants whose TG levels were <400 mg/dl.^23^ Dyslipidemia was defined as currently using lipid‐lowering drugs, and/or TC ≥240 mg/dl, and/or TG ≥200 mg/dl, and/or LDL‐C ≥160 mg/dl, and/or HDL‐C <40 mg/dl after fasting for at least 10 h.^24^ Body mass index (BMI) was calculated as weight (kg)/height (m)^2^.

Other known or suspected risk factors for CVD, including demographic characteristics, lifestyle, medical history of chronic diseases, and family history of CVD, were collected using structured questionnaires face to face at each visit by trained data investigators. Age (in years), smoking status (never smoker, former smoker, current smoker), drinking status (current drinker or not), educational level (no education history, primary school, middle school, high school, college or above), monthly household income per capita (<300, 300–500, 500–800, 800–1200, 1200–2000, ≥2000 Chinese yuan [CNY]/month), and physical activity level (whether or not the ideal level was reached during the previous 12 months) were assessed with standardized questionnaires. The definition of ideal physical activity level was at least 150 min of moderate‐intensity physical activity or at least 75 min of vigorous‐intensity physical activity per week, which was recommended by World Health Organization.^25^ Ideal vegetable and fruit intake was defined as at least 500 g per day according to the dietary guidelines for Chinese residents.^26^

### 2.4 Outcome measures

Medical and death records were collected from hospital records and local public health department to obtain information on disease status and vital outcome events during the follow‐up visit. Information from electrocardiogram, 2D echocardiography, computed tomography scan, coronary arteriography, or cardiovascular biomarkers, etc. was used to ascertain the final diagnosis. A study‐wide end point assessment committee at Fuwai Hospital reviewed and verified the diagnosis. CVD death included any death from CVD during the follow‐up period. According to the *International Classification of Diseases, 10th Revision*, I00 to I99, I20 to I25, I21, and I60 to I69 were codes for CVD death, ischemic heart disease, acute MI, and stroke, respectively. In this study, CVD was defined as presence of acute MI, unstable angina, nonfatal stroke, heart failure, and death from any CVD causes.^27^ The onset time of CVD was identified as the self‐reported date of first diagnosis or the date of CVD death.

CHD was defined as a diagnosis of acute MI, unstable angina, or death due to CHD. Acute MI was identified as a change in biochemical markers of myocardial necrosis accompanied by any of the following four characteristics: ischemic symptoms, pathological Q waves, ST‐segment elevation or depression, or coronary intervention.^28^ Unstable angina was identified as angina pectoris that changes or worsens. CHD death was defined as fatal events secondary to CHD, including fatal MI or other coronary deaths. Stroke included clinical signs and symptoms of subarachnoid or intracerebral hemorrhage or cerebral infarction, which were rapidly developing signs of focal (or global) disturbances in cerebral function lasting >24 h without an apparent nonvascular cause.

### 2.5 Statistical analysis

Continuous variables were expressed as mean ± SD or median values with the interquartile range. Categorical variables were expressed as numbers (percentages). Person‐years of follow‐up were calculated as the interval from the date of the second visit to the date of the final follow‐up interview, death, or the occurrence of incident CVD, whichever occurred first. We applied *t* test to compare values of normal distributions, Wilcoxon's rank‐sum test to compare data of abnormal distributions, and the chi‐square test to compare the frequencies of categorical variables between groups.

Cox proportional hazards regression model was used to estimate the association of FBG with CVD outcome as hazard ratios (HR) and 95% confidence interval (CI). Model 1 adjusted for age and gender, and model 2 additionally adjusted for BMI, smoking and drinking status, physical activity and educational level, hypertension status, dyslipidemia, FBG at the first visit, region, urbanicity, income, and family history of CVD.

Sensitivity analysis was conducted to explore the influence of excluding CVD incidence in the first year of the follow‐up period, excluding development of diabetes ahead of the onset of CVD during follow‐up period, including vegetable and fruit intake at the second visit into the multivariate‐adjusted model, taking cohort source as a stratum in the model to address potential effect variation between two cohorts. All analyses were performed using SAS, version 9.4 (SAS Institute Inc., Cary, North Carolina), and the statistical significance was set at a two‐tailed *p* value <.05.

## 3 RESULTS

### 3.1 Descriptive analysis

Table 1 shows characteristics of the 12 145 participants according to FBG status at the first visit. Participants with IFG (*n* = 2475) were more likely to be older and more obese, with high blood pressure, TC, TG, and LDL‐C levels and low HDL‐C levels, and they were less likely to have ideal physical activity and more likely to live in an urban area (Table 1). Compared with participants who were absent of the second visit and lost to follow‐up, those followed up tended to be slightly younger; less educated; have lower level of glucose, SBP, and TG; and more likely to have ideal physical activity and live in a rural area or north of China (Appendix S1: Table S1). The first‐visit characteristics among participants of six categories according to FBG of the first two surveys are provided in Appendix S1: Table S2. Participants with persistent IFG or progressing to DM from NFG/IFG rarely held a college degree or above, and tended to have higher BMI, blood pressure, TC, TG, and LDL‐C levels and lower HDL‐C levels.

**TABLE 1:** Characteristics of the study participants

Characteristics	NFG (*n* = 9670)	IFG (*n* = 2475)	*t*/*χ* ^2^	*p*
Age (years)	47.05 ± 8.45	50.16 ± 9.35	16.00	<.001
Male, *n* (%)	4453 (46.05)	1200 (48.48)	4.70	.030
BMI (kg/m^2^)	23.24 ± 3.39	24.22 ± 3.84	12.54	<.001
Smoking status, *n* (%)			20.68	<.001
Never	6166 (63.84)	1603 (64.79)		
Former	519 (5.37)	184 (7.44)		
Current	2973 (30.78)	687 (27.77)		
Alcohol drinkers, *n* (%)	2331(24.14)	679 (27.49)	11.84	.001
Education, *n* (%)			22.73	<.001
No education history	931 (9.73)	292 (11.95)		
Primary school	2915 (30.47)	800 (32.75)		
Middle school	2853 (29.82)	707 (28.94)		
High school	1997 (20.88)	436 (17.85)		
College or above	870 (9.09)	208 (8.51)		
Income, *n* (%)			7.93	.160
<300 CNY/month	4269 (44.65)	1145 (46.89)		
300–500 CNY/month	2124 (22.22)	499 (20.43)		
500–800 CNY/month	1866 (19.52)	485 (19.86)		
800–1200 CNY/month	858 (8.97)	196 (8.03)		
1200–2000 CNY/month	306 (3.20)	76 (3.11)		
≥2000 CNY/month	137 (1.43)	41 (1.68)		
Family history of CVD, *n* (%)	1414 (14.62)	215 (8.69)	59.78	<.001
Ideal physical activity, *n* (%)	5376 (57.48)	1235 (51.52)	27.51	<.001
Ideal vegetable and fruit intake at the second visit, *n* (%)	4706 (49.01)	1160 (47.21)	2.52	.113
North, *n* (%)	4933 (51.01)	1210 (48.89)	3.56	.059
Urban, *n* (%)	3681 (38.07)	1047 (42.30)	14.88	<.001
Glucose at the first visit (mg/dl)	86.61 ± 8.74	107.56 ± 6.45	111.73	<.001
Glucose at the second visit (mg/dl)	91.93 ± 18.55	105.35 ± 30.64	27.59	<.001
SBP (mmHg)	121.00 ± 18.37	127.16 ± 19.98	14.62	<.001
DBP (mmHg)	77.60 ± 10.99	80.38 ± 10.87	11.26	<.001
Dyslipidemia, *n* (%)	2711 (28.11)	1019 (41.31)	160.55	<.001
TC (mg/dl)	184.36 ± 35.61	196.30 ± 39.06	14.58	<.001
TG (mg/dl)^a^	103.60 (76.10, 144.10)	122.90 (86.20, 180.10)	14.44^b^	<.001
HDL‐C (mg/dl)	52.17 ± 13.04	50.76 ± 13.88	4.74	<.001
LDL‐C (mg/dl)	108.37 ± 31.76	116.53 ± 35.30	10.95	<.001

*Note*: Data are presented as the mean ± SD for continuous variables and as *n* (%) for categorical variables.

Abbreviations: BMI, body mass index; CNY, Chinese yuan; CVD, cardiovascular disease;DBP, diastolic blood pressure; HDL‐C, high‐density lipoprotein cholesterol; IFG, impaired fasting glucose (100–125 mg/dl); LDL‐C, low‐density lipoprotein cholesterol; NFG, normal fasting glucose (50–99 mg/dl); SBP, systolic blood pressure; TC, total cholesterol; TG, triglyceride.

^a^ Median (Q1, Q3).

^b^ *Z* statistic of Wilcoxon's rank‐sum test.

During a median follow‐up of 5.5 years, 373 incident cases of CVD (216 stroke and 139 CHD cases) were identified. The incidence density (per 1000 person‐years) of CVD was 4.87 in participants remaining NFG, 5.21 in those progressing from NFG to IFG, 11.99 in those progressing from NFG to DM, 5.22 in those reverting from IFG to NFG, 9.28 in those remaining IFG, and 10.28 in those progressing from IFG to DM (Table 2).

**TABLE 2:** Changes of fasting glucose levels and the risk of cardiovascular disease (CVD), stroke, and coronary heart disease (CHD)

The first visit	Characteristics	Fasting blood glucose at the second visit		
		NFG	IFG	DM
NFG	Counts	7825	1425	420
	CVD			
	Events	207	41	27
	Incidence density (per 1000 person‐years)	4.87	5.21	11.99
	Age, gender‐adjusted HR (95% CI)	Ref^b^	0.998 (0.714, 1.395)	2.237 (1.497, 3.341)
	Multivariate‐adjusted HR (95% CI)^a^		0.970 (0.687, 1.370)	1.723 (1.122, 2.645)
	Stroke			
	Events	121	24	18
	Incidence density (per 1000 person‐years)	2.84	3.04	7.91
	Age, gender‐adjusted HR (95% CI)	Ref^b^	1.001 (0.646, 1.552)	2.513 (1.531, 4.124)
	Multivariate‐adjusted HR (95% CI)^a^		0.991 (0.635, 1.547)	1.828 (1.067, 3.133)
	CHD	80	14	9
	Events			
	Incidence density (per 1000 person‐years)	1.87	1.77	3.94
	Age, gender‐adjusted HR (95% CI)	Ref^b^	0.887 (0.502, 1.565)	1.953 (0.980, 3.891)
	Multivariate‐adjusted HR (95% CI)^a^		0.826 (0.453, 1.505)	1.546 (0.756, 3.162)
IFG	Counts	1312	749	414
	CVD			
	Events	37	38	23
	Incidence density (per 1000 person‐years)	5.22	9.28	10.28
	Age, gender‐adjusted HR (95% CI)	0.874 (0.615, 1.243)	1.405 (0.991, 1.992)	1.640 (1.064, 2.527)
	Multivariate‐adjusted HR (95% CI)^a^	1.178 (0.760, 1.826)	1.792 (1.141, 2.816)	1.946 (1.120, 3.381)
	Stroke			
	Events	22	17	14
	Incidence density (per 1000 person‐years)	3.09	4.14	6.20
	Age, gender‐adjusted HR (95% CI)	0.890 (0.564, 1.404)	1.074 (0.644, 1.792)	1.677 (0.962, 2.924)
	Multivariate‐adjusted HR (95% CI)^a^	1.177 (0.667, 2.075)	1.308 (0.691, 2.474)	2.135 (1.056, 4.315)
	CHD			
	Events	11	18	7
	Incidence density (per 1000 person‐years)	1.54	4.36	3.09
	Age, gender‐adjusted HR (95% CI)	0.694 (0.368, 1.307)	1.797 (1.071, 3.016)	1.323 (0.609, 2.873)
	Multivariate‐adjusted HR (95% CI)^a^	0.851 (0.391, 1.851)	2.059 (1.014, 4.183)	1.094 (0.400, 2.993)

Abbreviations: BMI, body mass index; CI, confidence interval; DM, diabetes mellitus (≥126 mg/dl and/or using hypoglycemic drugs, and/or a self‐reported history of DM); FBG, fasting blood glucose; HR, hazard ratio; IFG, impaired fasting glucose (100–125 mg/dl); NFG, normal fasting glucose (50–99 mg/dl).

^a^ NFG both at the first and the second visit as reference.

^b^ Adjusted for age, gender, BMI, FBG at the first visit, smoking status (never smoker, former smoker, current smoker), drinking status (yes vs. no), physical activity, hypertension status (yes vs. no), dyslipidemia (yes vs. no), region (south vs. north), area (urban vs. rural), education, income, and family history of CVD.

### 3.2 Association of changes in FBG with CVD

As shown in Table 2, compared with persistent NFG, persistent IFG was associated with a 79.2% (HR 1.792; 95% CI 1.141, 2.816) higher risk of CVD incidence in the multivariate‐adjusted model. Moreover, participants who progressed from NFG to DM and progressed from IFG to DM had a higher risk of CVD than those remaining NFG, and the multivariate‐adjusted HR (95% CI) was 1.723 (1.122, 2.645), and 1.946 (1.120, 3.381), respectively. No significant association for CVD was found among those progressing from NFG to IFG (HR 0.970; 95% CI 0.687, 1.370), reverting from IFG to NFG (HR 1.178; 95% CI 0.760, 1.826).

In the analysis of changes in FBG and risk of stroke and CHD, progression from NFG or IFG to DM increased the risk of stroke than persistent NFG, and the multivariate‐adjusted HR (95% CI) was 1.828 (1.067, 3.133), and 2.135 (1.056, 4.315), respectively. Persistent IFG had a higher risk of CHD (HR 2.059; 95% CI 1.014, 4.183), whereas it was not significantly associated with stroke risk (HR 1.308; 95% CI 0.691, 2.474).

**FIGURE 3:**
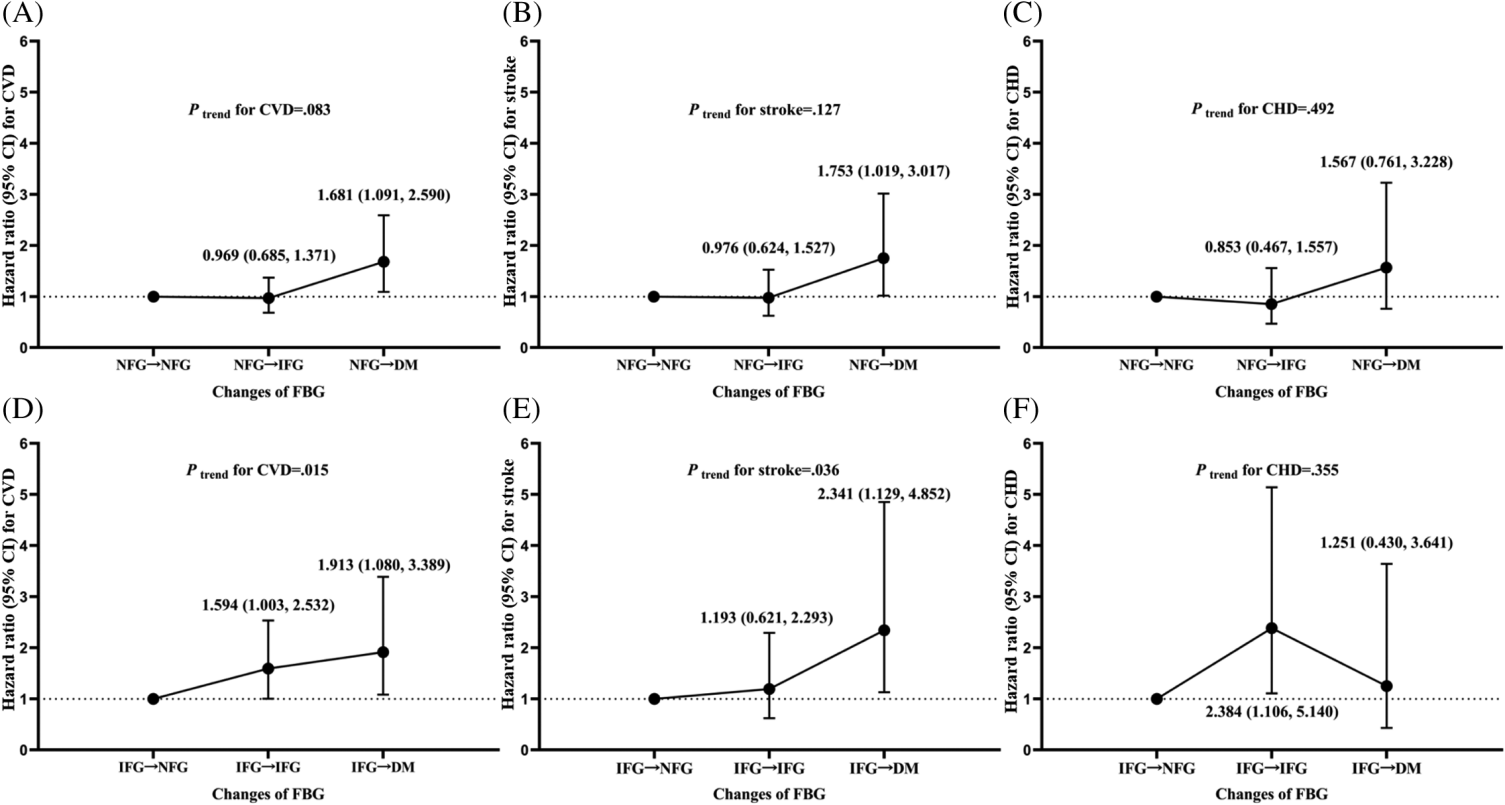
Trends and multivariate‐adjusted HRs of associations between changes of FBG and risk of cardiovascular disease (CVD), stroke, and coronary heart disease (CHD) in participants stratified by FBG status at the first visit ((a)–(c): persistent NFG was regarded as reference; (d)–(f): reversion from IFG to NFG was regarded as reference). Adjusted for age, gender, BMI, FBG at the first visit, smoking status (never smoker, former smoker, current smoker), drinking status (yes vs. no), physical activity, hypertension status (yes vs. no), dyslipidemia (yes vs. no), region (south vs. north), area (urban vs. rural), education, income, and family history of CVD. BMI, body mass index; CI, confidence interval; DM, diabetes mellitus (≥126 mg/dl and/or using hypoglycemic drugs, and/or a self‐reported history of DM); FBG, fasting blood glucose; HR, hazard ratio; IFG, impaired fasting glucose (100–125 mg/dl); NFG, normal fasting glucose (50–99 mg/dl)

In addition, there was a rising trend of CVD and stroke risk of subjects reverting from IFG to NFG, remaining IFG, and progressing from IFG to DM (*P*_trend_, .015 and .036, respectively). Compared with participants reverting from IFG to NFG, those remaining IFG had a higher risk of CVD (HR 1.594; 95% CI 1.003, 2.532), and those progressing from IFG to DM had a higher risk of CVD (HR 1.913; 95% CI 1.080, 3.389) and stroke (HR 2.341; 95% CI 1.129, 4.852) after adjustment for confounding factors (Figure 3). Although there was no significant rising trend of CHD risk among those groups, our study found persistent IFG increased the risk of CHD (HR 2.384; 95% CI 1.106, 5.140).

### 3.3 Sensitivity analysis

After excluding participants with the diagnosis of CVD within the first year of the follow‐up period (*n* = 39), persistent IFG, progression from NFG to DM, and progression from IFG to DM in comparison with persistent NFG still significantly increased the CVD risk. Multivariate‐adjusted HRs (95% CI) for CVD did not obviously change in those groups of FBG status, which were 1.746 (1.078, 2.827), 1.779 (1.137, 2.785), and 2.012 (1.124, 3.604), respectively (Appendix S1: Table S3). Moreover, as shown in Appendix S1: Table S4 – S6, the results remained robust after excluding participants developing diabetes ahead of the onset of CVD during follow‐up period (*n* = 202), adding vegetable and fruit intake into the covariates, or using the cohort‐stratified Cox regression model to estimate HR and 95% CI.

## 4 DISCUSSION

In this prospective cohort study, long‐standing IFG and progression to DM from NFG and IFG were associated with an elevated risk of CVD. Although several studies indicated that prediabetes significantly increased CVD risk only when it was defined by IGT or combined criteria,^29^, ^30^ our study revealed that in consideration of blood glucose transition, IFG‐defined prediabetes, which was monitored cheaply and conveniently, could detect cardiovascular impairment. Our results further indicated once IFG was detected, early glycemic control might slow the progression of macrovascular complications related with hyperglycemia.

Meta‐analysis showed IFG based on single measurement was associated with an increased CVD risk, whereas several studies reported inconsistent results.^31^, ^32^, ^33^ For instance, the Multi‐Ethnic Study of Atherosclerosis (MESA) showed that subjects with IFG did not have an increased risk for CVD events,^32^ which might underestimate the actual effect of IFG in contrast to NFG on CVD for regarding low glucose as part of the reference group.^34^ However, our project previously reported that IFG was still not associated with risk of atherosclerotic CVD when low fasting glucose (LFG) was separately considered.^33^ Notably, the association between IFG and CVD might be attenuated in these researches, because of the condition that a large proportion of IFG reverted to NFG during the follow‐up.^35^ Hence, multiple measurements are needed to evaluate the effect of changes of FBG on CVD.

In line with previous studies involving FBG transition, we drew steady results that newly discovered diabetes increased CVD risk.^13^, ^14^ In addition, several studies showed persistent IFG did not significantly increase CVD risk,^13^, ^14^, ^16^ and a high risk of cardiovascular mortality in subjects with IFG was on account of conversion to diabetes.^15^ Inconsistent with this evidence, our results showed that long‐standing rather than newly discovered IFG was associated with an increased risk of CVD, demonstrating that IFG itself played the role in cardiovascular health hazards, and it might take time to influence cardiovascular health. The effect of persistent IFG on cardiovascular mortality might be underestimated due to two reasons in the Hoorn Study.^15^ First, the NFG group was classified according to single measurement, in which some subjects might develop diabetes before the second visit. Second, subjects progressing to diabetes were separated from the IFG group, whereas those reverting to NFG were ignored. Moreover, the sample size of the aforementioned studies was limited and confounding factors were not completely controlled.^15^, ^16^ Furthermore, our study extended the findings of the Kailuan Study,^17^ suggesting subjects reverting from IFG to NFG had a lower risk of CVD not just than those progressing from IFG to DM but also than those with persistent IFG. Therefore, early management of IFG would be of great value in preventing CVD in Chinese population.

Potential mechanisms of hyperglycemia in increasing CVD risk are as follows. First, evidence showed that the transition from NFG to prediabetes in subjects was associated with increased weight and insulin resistance and reduced endogenous insulin secretion.^36^ Second, further dysfunctions in prediabetes include increased lipolysis, decreased endogenous levels of glucagon‐like peptide 1, and impaired postprandial suppression of glucagon secretion by the alpha cells of the pancreas.^8^ Third, hyperglycemia may contribute to atherosclerosis through increase of oxidative stress, activation of thrombosis, and changes of epigenetics.^37^, ^38^ Furthermore, intestinal physical barrier abnormality may also play a role in the association between disturbance of carbohydrate metabolism and occurrence of adverse cardiovascular events.^39^

Though both IGT and IFG were related to the increased risk of CVD,^11^ their pathophysiological mechanisms might be different. IGT is associated with more skeletal muscle (peripheral) insulin resistance than IFG. IFG is characterized by hepatic insulin resistance and excessive endogenous glucose production. Individuals with IFG plus IGT exhibit defects in both peripheral and hepatic insulin sensitivity as well as a progressive loss of beta cell function over time.^40^ Participants with IFG plus IGT or with isolated IGT were reported to have higher risks of CVD than those with isolated IFG.^11^, ^41^, ^42^ Lifestyle interventions tend to improve endothelial function and insulin resistance, leading to a decreased incidence of CVD in prediabetic population.^43^ For example, in the Da Qing Diabetes Prevention Study 6‐year lifestyle intervention reduced the incidence of CVD after 20–30 years of follow‐up.^44^ Intervention to prevent CVD should target the abnormality of glucose as early as possible.

Several strengths of this study are as follows. We used data from a nationwide, prospective cohort from 14 provinces with long‐time multiple rounds of follow‐up survey.^19^ Furthermore, owing to a nonlinear relationship reported between blood glucose and CVD risk,^34^, ^45^ we excluded subjects with FBG <50 mg/dl when we defined normoglycemia, according to the diagnostic criteria of hypoglycemia for the population without diabetes in China,^21^ which may contribute to a reliable evaluation of the relationship between different FBG status and CVD.

However, the limitations of our research warrant consideration: first, postprandial glucose, 2‐h plasma glucose during 75‐g oral glucose tolerance test, and HbA1c were not measured, which are also important for the development of atherosclerosis and can reflect prediabetes according to different criteria and minimize the possibility of misclassifying prediabetes.^46^ However, FBG is a cost‐effective method of detecting blood glucose level and could be widely applied for CVD risk stratification. Further studies are warranted to provide additional evidence on the association of different phenotypes of prediabetes with CVD. Second, though plasma is recommended for testing FBG levels, which is lower by 1.15% when using serum, the difference may not be physiologically relevant.^1^, ^47^ Besides, all the samples were stored at −80°C after transportation under cold chain conditions, which minimized the decline in glucose levels. Third, because of the limited number of subjects with hypoglycemia, the analysis of the effect of changes in hypoglycemia on CVD risk could not be conducted. Moreover, the small number of stroke and CHD events caused unstable associations of persistent IFG with stroke and CHD, yet our results indicated the associations of persistent IFG were stronger for CHD than for stroke. What is more, the hyperglycemic period was detected by the follow‐up interval of 8 years, so the association between hyperglycemic periods of different lengths and CVD risk could not be more accurately detected. In addition, our central laboratories passed the quality control in the Lipid Standardization Program of the US Centers for Disease Control and Prevention, and the methods and instruments of serum glucose and lipids measurement were consistent at both visits. Thus, the influence of systematic measurement bias was probably limited due to the stringent quality control of blood sample measurement. Finally, there was also a possibility of unmeasured confounding in this prospective cohort study.

## 5 CONCLUSIONS

Long‐standing prediabetes status defined by IFG and progressing from NFG/IFG to DM within the interval of 8 years had an increased risk of developing CVD. Emphasis on surveillance and management of FBG for prediabetes is indispensable for the primary prevention of CVD. Further well‐designed studies are needed to examine the effect of different prediabetes phenotypes and prediabetes duration on health outcome.

## FUNDING INFORMATION

The National Key Research and Development Program of China, Grant/Award Numbers: 2018YFE0115300, 2017YFC0211703; the Chinese Academy of Medical Sciences Innovation Fund for Medical Sciences, Grant/Award Numbers: 2021‐I2M‐1‐010, 2017‐I2M‐1‐004, and 2019‐I2M‐2‐003; the National Natural Science Foundation of China, Grant/Award Numbers: 82030102, 12126602; the Research Unit of Prospective Cohort of Cardiovascular Diseases and Cancers, Chinese Academy of Medical Sciences, Grant/Award Numbers: 2019RU038.

## DISCLOSURE

The authors have no conflicts of interest to disclose.

## Supporting information


**Appendix S1.** Supporting information.
