# Supplementary material for: Changes in fasting blood glucose status and incidence of cardiovascular disease: The China‐PAR project
Source: J Diabetes. 2023 Jan 13;15(2):110–20. doi: 10.1111/1753-0407.13350 (PMC9934960; doi:10.1111/1753-0407.13350)
Supplement: Supplementary file 1 — Appendix S1. Supporting information. [file JDB-15-110-s001.docx]

**Contents**:

**Supplemental Method**: Blood specimens' measurement

**Supplemental Table 1**. Comparison of characteristics between participants followed up and those absent of the second visit and lost to follow-up.

**Supplemental Table 2**. The first-visit characteristics among participants of six categories according to FBG of the first two surveys.

**Supplemental Table 3**. Changes of fasting glucose levels and risk of CVD after excluding participants developing CVD within the first year of follow-up.

**Supplemental Table 4**. Changes of fasting glucose levels and risk of CVD after excluding development of diabetes ahead of the onset of CVD during the follow-up period.

**Supplemental Table 5**. Changes of fasting glucose levels and risk of CVD after including vegetables and fruits intake at the second visit into the model.

**Supplemental Table 6**. Changes of fasting glucose levels and risk of CVD after taking cohort source as a stratum in the model.

**Blood specimens' measurement**

Blood specimens of participants at baseline examination and the follow-up visits from InterASIA were processed at the field centers and shipped to the central clinical laboratory at the Department of Population Genetics at Fuwai Hospital, where the specimens were stored at -80°C until laboratory assays could be performed. As for China MUCA-1998, those at baseline were measured with the same protocol and quality control program, though measurements were conducted in the central laboratory at Fuwai Hospital or Guangdong Cardiovascular Institute, as well as local laboratories respectively, and those at the first follow-up visit were all measured in the central laboratory at Fuwai Hospital.

Our central laboratory has participated in the Lipid Standardization Program of the US Centers for Disease Control and Prevention since the mid-1980s. Local laboratories in the China MUCA-1998 were under stringent quality control from the central laboratory. Unified quality control serum samples were used for external and internal quality assessment and measurement results of all laboratories were needed to be reported periodically.

Furthermore, the methods and instruments of serum glucose and lipids measurement were consistent at both visits.

**Supplemental Table 1**. Comparison of characteristics between participants followed up and those absent of the second visit and lost to follow-up.

| Characteristics | include (n=12145) | exclude (n=9488) | t/χ^2^ | *P* |
| --- | --- | --- | --- | --- |
| Age, years | 47.68±8.73 | 49.41±9.89 | 13.62 | <0.001 |
| Male, n (%) | 5653 (46.55) | 4590 (48.38) | 7.16 | 0.007 |
| BMI, kg/m² | 23.44±3.51 | 23.72±3.50 | 5.77 | <0.001 |
| Smoking status, n (%) |  |  | 15.74 | <0.001 |
| Never | 7769 (64.04) | 6004 (63.45) |  |  |
| Former | 703 (5.79) | 673 (7.11) |  |  |
| Current | 3660 (30.17) | 2785 (29.43) |  |  |
| Alcohol-drinkers, n (%) | 3010 (24.82) | 2296 (24.40) | 0.50 | 0.480 |
| Education, n (%) |  |  | 249.42 | <0.001 |
| No education history | 1223 (10.18) | 965 (10.48) |  |  |
| Primary school | 3715 (30.94) | 2294 (24.91) |  |  |
| Middle school | 3560 (29.64) | 2366 (25.70) |  |  |
| High school | 2433 (20.26) | 2377 (25.81) |  |  |
| College or above | 1078 (8.98) | 1206 (13.10) |  |  |
| Income, n (%) |  |  | 66.90 | <0.001 |
| <300 CNY/month | 5414 (45.11) | 3732 (40.56) |  |  |
| 300-500 CNY/month | 2623 (21.85) | 2346 (25.50) |  |  |
| 500-800 CNY/month | 2351 (19.59) | 1965 (21.36) |  |  |
| 800-1200 CNY/month | 1054 (8.78) | 781 (8.49) |  |  |
| 1200-2000 CNY/month | 382 (3.18) | 261 (2.84) |  |  |
| ≥2000 CNY/month | 178 (1.48) | 116 (1.26) |  |  |
| Family history of CVD, n (%) | 1629 (13.41) | 1117 (11.77) | 12.93 | <0.001 |
| Ideal physical activity, n (%) | 6611 (56.26) | 4068 (44.96) | 261.44 | <0.001 |
| North, n (%) | 6143 (50.58) | 4076 (42.96) | 124.13 | <0.001 |
| Urban, n (%) | 4728 (38.93) | 5828 (61.42) | 1078.83 | <0.001 |
| Glucose at the first visit, mg/dL | 90.88±11.86 | 96.39±25.62 | 20.85 | <0.001 |
| SBP, mmHg | 122.26±18.87 | 123.11±20.21 | 3.20 | 0.001 |
| DBP, mmHg | 78.17±11.02 | 78.09±11.67 | 0.53 | 0.593 |
| Dyslipidemia, n (%) | 3730 (30.80) | 3118 (34.57) | 33.58 | <0.001 |
| TC, mg/dL | 186.79±36.65 | 184.11±37.28 | 5.22 | <0.001 |
| TG, mg/dL ^✝^ | 107.00 (77.90, 150.40) | 110.50 (80.70, 156.00) | 4.86 ^‡^ | <0.001 |
| HDL-C, mg/dL | 51.88±13.22 | 50.41±13.47 | 7.94 | <0.001 |
| LDL-C, mg/dL | 109.99±32.66 | 107.78±33.12 | 4.79 | <0.001 |

*Note*: Data are presented as the mean ± SD for continuous variables and as n (%) for categorical variables.

✝: median (Q1, Q3); ‡: Z statistic of Wilcoxon's rank sum test.

Abbreviations: BMI, body mass index; SBP, systolic blood pressure; DBP, diastolic blood pressure; TC, total cholesterol; TG, triglyceride; HDL-C, high-density lipoprotein cholesterol; LDL-C, low-density lipoprotein cholesterol.

**Supplemental Table 2**. The first-visit characteristics among participants of six categories according to FBG of the first two surveys.

| Characteristics | NFG→NFG (n=7825) | NFG→IFG  (n=1425) | NFG→DM  (n=420) | IFG→NFG  (n=1312) | IFG→IFG  (n=749) | IFG→DM  (n=414) |
| --- | --- | --- | --- | --- | --- | --- |
| Age, years | 46.81±8.46 | 47.94±8.40 | 48.45±8.17 | 49.49±9.42 | 51.04±9.32 | 50.71±9.04 |
| Male, n (%) | 3531 (45.12) | 709 (49.75) | 213 (50.71) | 628 (47.87) | 373 (49.80) | 199 (48.07) |
| BMI, kg/m² | 22.96±3.24 | 24.04±3.64 | 25.64±3.83 | 23.52±3.39 | 24.44±3.52 | 26.05±4.92 |
| Smoking status, n (%) |  |  |  |  |  |  |
| Never | 5038 (64.47) | 894 (62.83) | 234 (55.71) | 849 (64.71) | 492 (65.78) | 262 (63.29) |
| Former | 410 (5.25) | 77 (5.41) | 32 (7.62) | 95 (7.24) | 55 (7.35) | 34 (8.21) |
| Current | 2367 (30.29) | 452 (31.76) | 154 (36.67) | 368 (28.05) | 201 (26.87) | 118 (28.50) |
| Alcohol-drinkers, n (%) | 1809 (23.14) | 401 (28.24) | 121 (28.81) | 354 (27.00) | 215 (28.78) | 110 (26.70) |
| Education, n (%) |  |  |  |  |  |  |
| No education history | 760 (9.82) | 128 (9.09) | 43 (10.31) | 154 (11.86) | 95 (12.86) | 43 (10.59) |
| Primary school | 2358 (30.46) | 434 (30.82) | 123 (29.50) | 423 (32.59) | 249 (33.69) | 128 (31.53) |
| Middle school | 2266 (29.27) | 446 (31.68) | 141 (33.81) | 381 (29.35) | 197 (26.66) | 129 (31.77) |
| High school | 1644 (21.24) | 273 (19.39) | 80 (19.18) | 220 (16.95) | 136 (18.40) | 80 (19.70) |
| College or above | 713 (9.21) | 127 (9.02) | 30 (7.19) | 120 (9.24) | 62 (8.39) | 26 (6.40) |
| Income, n (%) |  |  |  |  |  |  |
| <300 CNY/month | 3553 (45.94) | 531 (37.74) | 185 (44.15) | 658 (50.73) | 335 (45.52) | 152 (37.16) |
| 300-500 CNY/month | 1744 (22.55) | 295 (20.97) | 85 (20.29) | 244 (18.81) | 145 (19.70) | 110 (26.89) |
| 500-800 CNY/month | 1454 (18.80) | 324 (23.03) | 88 (21.00) | 250 (19.28) | 150 (20.38) | 85 (20.78) |
| 800-1200 CNY/month | 652 (8.43) | 165 (11.73) | 41 (9.79) | 93 (7.17) | 62 (8.42) | 41 (10.02) |
| 1200-2000 CNY/month | 222 (2.87) | 68 (4.83) | 16 (3.82) | 32 (2.47) | 33 (4.48) | 11 (2.69) |
| ≥2000 CNY/month | 109 (1.41) | 24 (1.71) | 4 (0.95) | 20 (1.54) | 11 (1.49) | 10 (2.44) |
| Family history of CVD, n (%) | 1073 (13.71) | 248 (17.40) | 93 (22.14) | 108 (8.23) | 59 (7.88) | 48 (11.59) |
| Ideal physical activity, n (%) | 3202 (42.27) | 594 (43.29) | 181 (44.58) | 573 (44.77) | 381 (52.70) | 208 (52.79) |
| Ideal vegetables and fruits intake at the second visit, n (%) | 3785 (48.71) | 713 (50.28) | 208 (50.24) | 626 (48.01) | 331 (44.55) | 203 (49.51) |
| North, n (%) | 3875 (49.52) | 782 (54.88) | 276 (65.71) | 633 (48.25) | 357 (47.66) | 220 (53.14) |
| Urban, n (%) | 2955 (37.76) | 581 (40.77) | 145 (34.52) | 512 (39.02) | 348 (46.46) | 187 (45.17) |
| Glucose at the first visit, mg/dL | 86.10±8.76 | 88.47±8.42 | 89.61±7.94 | 106.38±5.78 | 107.73±6.30 | 111.01±7.43 |
| Glucose at the second visit, mg/dL | 86.15±8.35 | 107.50±6.32 | 147.88±44.97 | 88.24±8.53 | 109.11±6.78 | 153.69±45.60 |
| SBP, mmHg | 119.94±17.88 | 124.34±19.07 | 129.43±21.24 | 125.05±19.42 | 129.08±20.12 | 130.37±20.74 |
| DBP, mmHg | 76.97±10.76 | 79.47±11.44 | 82.96±11.54 | 79.58±10.88 | 80.99±10.68 | 81.84±10.97 |
| Dyslipidemia, n (%) | 2053 (26.31) | 474(33.33) | 184 (43.91) | 485 (37.02) | 316 (42.42) | 218 (52.91) |
| TG, mg/dL ^✝^ | 101.70 (75.30, 139.00) | 111.00 (77.90, 159.65) | 129.50 (92.00, 190.00) | 115.00 (83.00, 169.00) | 123.40 (87.75, 174.00) | 147.70 (103.10, 217.30) |
| TC, mg/dL | 183.14±35.08 | 188.69±36.99 | 192.27±38.28 | 192.88±37.94 | 201.26±40.91 | 198.25±38.11 |
| HDL-C, mg/dL | 52.55±13.02 | 51.13±13.09 | 48.55±12.54 | 52.12±14.04 | 50.27±13.29 | 47.30±13.76 |
| LDL-C, mg/dL | 107.46±31.30 | 111.85±32.68 | 113.62±35.63 | 113.63±34.51 | 122.13±37.06 | 115.57±33.35 |

*Note*: Data are presented as the mean ± SD for continuous variables and as n (%) for categorical variables.

✝: median (Q1, Q3).

Abbreviations: BMI, body mass index; SBP, systolic blood pressure; DBP, diastolic blood pressure; TC, total cholesterol; TG, triglyceride; HDL-C, high-density lipoprotein cholesterol; LDL-C, low-density lipoprotein cholesterol; FBG, fasting blood glucose; NFG, normal fasting glucose (50-99 mg/dL); IFG, impaired fasting glucose (100-125 mg/dL); DM, diabetes mellitus (≥126 mg/dL and/or using hypoglycemic drugs, and/or a self-reported history of DM).

**Supplemental Table 3**. Changes of fasting glucose levels and risk of CVD after excluding participants developing CVD within the first year of follow-up.

| The first visit | Characteristics | Fasting blood glucose at the second visit | | |
| --- | --- | --- | --- | --- |
|  |  | NFG | IFG | DM |
| NFG |  |  |  |  |
|  | Counts | 7804 | 1420 | 418 |
|  | CVD | 186 | 36 | 25 |
|  | Events |  |  |  |
|  | Incidence density (per 1000 person-years) | 4.38 | 4.57 | 11.11 |
|  | Age, gender-adjusted HR (95% CI) | Ref ^‡^ | 0.973 (0.681, 1.391) | 2.311 (1.522, 3.509) |
|  | Multivariate-adjusted HR (95% CI) ^✝^ |  | 0.955 (0.660, 1.380) | 1.779 (1.137, 2.785) |
| IFG |  |  |  |  |
|  | Counts | 1308 | 744 | 412 |
|  | CVD | 33 | 33 | 21 |
|  | Events |  |  |  |
|  | Incidence density (per 1000 person-years) | 4.66 | 8.07 | 9.39 |
|  | Age, gender-adjusted HR (95% CI) | 0.868 (0.598, 1.259) | 1.354 (0.932, 1.968) | 1.668 (1.060, 2.624) |
|  | Multivariate-adjusted HR (95% CI) ^✝^ | 1.178 (0.740, 1.875) | 1.746 (1.078, 2.827) | 2.012 (1.124, 3.604) |

✝: adjusted for age, gender, BMI, FBG at the first visit, smoking status (never smoker, former smoker, current smoker), drinking status (yes vs no), physical activity, hypertension status (yes vs no), dyslipidemia (yes vs no), region (south vs north), area (urban vs rural), education, income and family history of CVD.

‡: NFG both at the first and the second visit as reference.

Abbreviations: BMI, body mass index; FBG, fasting blood glucose; NFG, normal fasting glucose (50-99 mg/dL); IFG, impaired fasting glucose (100-125 mg/dL); DM, diabetes mellitus (≥126 mg/dL and/or using hypoglycemic drugs, and/or a self-reported history of DM); CVD, cardiovascular disease.

**Supplemental Table 4**. Changes of fasting glucose levels and risk of CVD after excluding development of diabetes ahead of the onset of CVD during the follow-up period.

| The first visit | Characteristics | Fasting blood glucose at the second visit | | |
| --- | --- | --- | --- | --- |
|  |  | NFG | IFG | DM |
| NFG |  |  |  |  |
|  | Counts | 7774 | 1356 | 420 |
|  | Events | 205 | 38 | 27 |
|  | Incidence density (per 1000 person-years) | 4.86 | 5.08 | 12.00 |
|  | Age, gender-adjusted HR (95% CI) | Ref  ^‡^ | 0.970 (0.686, 1.372) | 2.245 (1.502, 3.354) |
|  | Multivariate-adjusted HR (95% CI) ^✝^ |  | 0.954 (0.668, 1.363) | 1.733 (1.128, 2.662) |
| IFG |  |  |  |  |
|  | Counts | 1292 | 687 | 414 |
|  | Events | 36 | 36 | 23 |
|  | Incidence density (per 1000 person-years) | 5.16 | 9.62 | 10.28 |
|  | Age, gender-adjusted HR (95% CI) | 0.866 (0.607, 1.237) | 1.446 (1.011, 2.068) | 1.647 (1.068 2.539) |
|  | Multivariate-adjusted HR (95% CI) ^✝^ | 1.160 (0.745, 1.807) | 1.830 (1.154, 2.901) | 1.955 (1.123, 3.405) |

✝: adjusted for age, gender, BMI, FBG at the first visit, smoking status (never smoker, former smoker, current smoker), drinking status (yes vs no), physical activity, hypertension status (yes vs no), dyslipidemia (yes vs no), region (south vs north), area (urban vs rural), education, income and family history of CVD.

‡: NFG both at the first and the second visit as reference.

Abbreviations: BMI, body mass index; FBG, fasting blood glucose; NFG, normal fasting glucose (50-99 mg/dL); IFG, impaired fasting glucose (100-125 mg/dL); DM, diabetes mellitus (≥126 mg/dL and/or using hypoglycemic drugs, and/or a self-reported history of DM); CVD, cardiovascular disease.

**Supplemental Table 5**. Changes of fasting glucose levels and risk of CVD after including vegetables and fruits intake at the second visit into the model.

| The first visit | Characteristics | Fasting blood glucose at the second visit | | |
| --- | --- | --- | --- | --- |
|  |  | NFG | IFG | DM |
| NFG |  |  |  |  |
|  | Counts | 7825 | 1425 | 420 |
|  | Events | 207 | 41 | 27 |
|  | Incidence density (per 1000 person-years) | 4.87 | 5.21 | 11.99 |
|  | Age, gender-adjusted HR (95% CI) | Ref  ^‡^ | 0.998 (0.714, 1.395) | 2.237 (1.497, 3.341) |
|  | Multivariate-adjusted HR (95% CI) ^✝^ |  | 0.974 (0.689, 1.375) | 1.663 (1.074, 2.573) |
| IFG |  |  |  |  |
|  | Counts | 1312 | 749 | 414 |
|  | Events | 37 | 38 | 23 |
|  | Incidence density (per 1000 person-years) | 5.22 | 9.28 | 10.28 |
|  | Age, gender-adjusted HR (95% CI) | 0.874 (0.615, 1.243) | 1.405 (0.991, 1.992) | 1.640 (1.064, 2.527) |
|  | Multivariate-adjusted HR (95% CI) ^✝^ | 1.164 (0.751, 1.806) | 1.815 (1.154, 2.856) | 1.920 (1.105, 3.337) |

✝: adjusted for age, gender, BMI, FBG at the first visit, smoking status (never smoker, former smoker, current smoker), drinking status (yes vs no), physical activity, hypertension status (yes vs no), dyslipidemia (yes vs no), region (south vs north), area (urban vs rural), education, income, ideal vegetables and fruits intake (yes vs no), and family history of CVD.

‡: NFG both at the first and the second visit as reference.

Abbreviations: BMI, body mass index; FBG, fasting blood glucose; NFG, normal fasting glucose (50-99 mg/dL); IFG, impaired fasting glucose (100-125 mg/dL); DM, diabetes mellitus (≥126 mg/dL and/or using hypoglycemic drugs, and/or a self-reported history of DM); CVD, cardiovascular disease.

**Supplemental Table 6**. Changes of fasting glucose levels and risk of CVD after taking cohort source as a stratum in the model.

| The first visit | Characteristics | Fasting blood glucose at the second visit | | |
| --- | --- | --- | --- | --- |
|  |  | NFG | IFG | DM |
| NFG |  |  |  |  |
|  | Counts | 7825 | 1425 | 420 |
|  | Events | 207 | 41 | 27 |
|  | Incidence density (per 1000 person-years) | 4.87 | 5.21 | 11.99 |
|  | Age, gender-adjusted HR (95% CI) | Ref  ^‡^ | 0.977 (0.698, 1.367) | 2.185 (1.462, 3.266) |
|  | Multivariate-adjusted HR (95% CI) ^✝^ |  | 0.951 (0.673, 1.343) | 1.653 (1.067, 2.560) |
| IFG |  |  |  |  |
|  | Counts | 1312 | 749 | 414 |
|  | Events | 37 | 38 | 23 |
|  | Incidence density (per 1000 person-years) | 5.22 | 9.28 | 10.28 |
|  | Age, gender-adjusted HR (95% CI) | 0.899 (0.631, 1.280) | 1.439 (1.013, 2.043) | 1.577 (1.014, 2.453) |
|  | Multivariate-adjusted HR (95% CI) ^✝^ | 1.131 (0.727, 1.757) | 1.746 (1.107, 2.754) | 1.794 (1.017, 3.165) |

✝: adjusted for age, gender, BMI, FBG at the first visit, smoking status (never smoker, former smoker, current smoker), drinking status (yes vs no), physical activity, hypertension status (yes vs no), dyslipidemia (yes vs no), region (south vs north), area (urban vs rural), education, income, ideal vegetables and fruits intake (yes vs no), and family history of CVD.

‡: NFG both at the first and the second visit as reference.

Abbreviations: BMI, body mass index; FBG, fasting blood glucose; NFG, normal fasting glucose (50-99 mg/dL); IFG, impaired fasting glucose (100-125 mg/dL); DM, diabetes mellitus (≥126 mg/dL and/or using hypoglycemic drugs, and/or a self-reported history of DM); CVD, cardiovascular disease.
